# PREDICTORS OF SHORTNESS OF BREATH DURING STAIR CLIMB IN COMMUNITY-DWELLING INDIVIDUALS WITH COPD

**DOI:** 10.1093/geroni/igz038.984

**Published:** 2019-11-08

**Authors:** Shweta Gore

**Affiliations:** MGH Institute of Health Professions, Boston, Massachusetts, United States

## Abstract

Dyspnea is the primary and most disabling symptoms seen in chronic obstructive pulmonary disease (COPD). Primary pathophysiological changes such as dynamic hyperinflation have been associated with the etiology of dyspnea in chronic obstructive pulmonary disease (COPD). However, since the experience of dyspnea “derives from interactions among multiple physiological, psychosocial and environmental factors”, a single correlate to accurately predict dyspnea has not been established. The purpose of this study was to identify factors that could predict shortness of breath during stair climb (SOB-SC) in community dwelling adults with COPD. We hypothesized that physical activity and muscle strength would significantly predict SOB-SC. Individuals with COPD who participated in the National Health and Nutrition Examination Survey (NHANES) between years 1999-2002 were selected for this study. Participants were excluded if they had significant mobility limitations. Socioeconomic, demographic variables, and clinical variables including BMI, physical activity, comorbidities, muscle strength, ankle brachial index, waist circumference and inflammatory markers were extracted. Logistic regression models were plotted with SOB-SC as the categorical dependent variable after assessing for collinearity using the forced-entry method. Individuals with COPD had a significantly greater proportion of SOB-SC (χ = 134.87, p < 0.001). Larger waist circumference (p = 0.002, CI = 0.04 -0.13), presence of cardiovascular disease (p = 0.001, CI = 0.76 -2.37) and Caucasian race were found to significantly predict SOB-SC after controlling for covariates. This study reinforces the importance of screening for cardiovascular disease and lifestyle modification in this population subgroup. Future studies examining differences in COPD severity are needed.

